# Distribution of algal aggregates under summer sea ice in the Central Arctic

**DOI:** 10.1007/s00300-014-1634-3

**Published:** 2014-12-17

**Authors:** Christian Katlein, Mar Fernández-Méndez, Frank Wenzhöfer, Marcel Nicolaus

**Affiliations:** 1 Alfred-Wegener-Institut Helmholtz-Zentrum für Polar- und Meeresforschung, Bussestr. 24, 27570 Bremerhaven, Germany; 2 Max Planck Institute for Marine Microbiology, 28359 Bremen, Germany

**Keywords:** Sea ice algae, Algal assemblages, Size distribution, *Melosira arctica* filaments, Image processing, Remotely operated vehicle

## Abstract

The sea ice cover of the Arctic Ocean has changed dramatically in the last decades, and the resulting consequences for the sea-ice-associated ecosystem remain difficult to assess. Algal aggregates underneath sea ice are of great importance for the ice-associated ecosystem and the pelagic-benthic coupling. However, the frequency and distribution of their occurrence is not well quantified. During the IceArc expedition (ARK-27/3) of RV Polarstern in late summer 2012, we observed different types of algal aggregates floating underneath various ice types in the Central Arctic basins. We investigated the spatial distribution of ice algal aggregates and quantified their biomass, using under-ice image surveys obtained by an upward-looking camera on a remotely operated vehicle. On basin scale, filamentous aggregates of *Melosira arctica* are more frequently found in the inner part of the Central Arctic pack ice, while rounded aggregates mainly formed by pennate diatoms are found closer to the ice edge, under melting sea ice. On the scale of an ice floe, the distribution of algal aggregates in late summer is mainly regulated by the topography of the ice underside, with aggregates accumulating in dome-shaped structures and at the edges of pressure ridges. The average biomass of the aggregates from our sites and season was 0.1–6.0 mg C m^−2^. However, depending on the approach used, differences in orders of magnitude for biomass estimates may occur. This highlights the difficulties of upscaling observations and comparing results from surveys conducted using different methods or on different spatial scales.

**Electronic supplementary material:**

The online version of this article (doi:10.1007/s00300-014-1634-3) contains supplementary material, which is available to authorized users.

## Introduction

The Arctic Ocean has changed dramatically in recent decades. Changes of physical processes in the climate system—such as decreased sea ice extent (Serreze et al. 2007) and thickness (Haas et al. 2008; Kwok and Rothrock 2009), the trend from multi-year to younger first-year sea ice (Maslanik et al. 2007), a longer melt season (Markus et al. 2009), increased melt-pond coverage (Roesel and Kaleschke 2012), and increased light transmittance through the ice (Nicolaus et al. 2012)—are affecting the sea ice ecosystem (Arrigo et al. 2008; Lee et al. 2011; Arrigo 2014). Assessing the consequences of these changes in sea ice ecosystems is difficult, as observations in the ice-covered Arctic Ocean are technically challenging, and we are lacking a comprehensive baseline—especially in the central basins—to make the effects of change apparent.

Sea ice harbors a complex diversity of life, both in its brine channels and associated with its ice-water interface, which is strongly influenced by the physical conditions present (Horner et al. 1992; Legendre et al. 1992; Krembs et al. 2002; Mundy et al. 2005; Quillfeldt et al. 2009). Sea ice algae play an important role in the sea ice ecosystem, supporting a substantial fraction of total primary productivity (Gosselin et al. 1997), potentially seeding the under-ice phytoplankton bloom in spring (Wassmann and Reigstad 2011), and providing an important food source for the zooplankton (Leu et al. 2010, 2011). Changed melting conditions and increased light availability are also expected to affect the life conditions of sea ice algae (Lee et al. 2011; Leu et al. 2010). When ice algae are released to the water column because of ice melt, they are inherently prone to aggregation due to the high production of transparent exopolymers (Riebesell et al. 1991; Krembs et al. 2011). Aggregation can then lead to either a rapid sedimentation of biomass on the seafloor (Boetius et al. 2013) or a prolonged suspension underneath the ice (Assmy et al. 2013) due to oxygen entrapment within the aggregates (Fernández-Méndez et al. 2014). In this paper, the term “algal aggregates” refers to macroscopic (>1 cm) mostly free-floating aggregations mainly formed by typical sea-ice-associated algae such as those described by Fernández-Méndez et al. (2014). These aggregates have previously been described in the literature using various names such as sub-ice assemblages, algal filaments or aggregations (Nansen 1906; Melnikov and Bondarchuk 1987; Horner et al. 1992; Gutt 1995). Despite the long history of observations of algal aggregates under Arctic sea ice, little is known about the factors controlling their spatial distribution on both floe and basin scales.

Observations of ice algal aggregates are sparse, and usually cover only a small spatial range, as it is a great challenge to gather spatial datasets underneath sea ice. Up to now, most studies have been based on diving operations (Melnikov and Bondarchuk 1987; Syvertsen 1991; Melnikov 1997; Poulin et al. 2014; Glud et al. 2014). The increased use of remotely operated vehicles (ROVs) in polar regions, along with the recent advances in digital underwater imaging, has now enabled us to perform detailed observations of under-ice environments on a larger spatial scale (Gutt 1995; Werner and Lindemann 1997; Perovich et al. 1998; Ambrose et al. 2005; Gradinger and Bluhm 2010; Nicolaus and Katlein 2013).

The objective of this paper is to quantify the amount and distribution of algal aggregates underneath Arctic summer sea ice on floe and basin scales using ROV surveys. The spatial distribution of aggregate abundance is analyzed as a function of the physical properties of the sea ice habitat. In addition, patchiness of the distribution as well as geometric properties of under-ice aggregates is investigated. Finally, different approaches for estimation of aggregate biomass are discussed to evaluate uncertainties and recommend procedures for future work.

## Materials and methods

### Remotely operated vehicle (ROV) observations

**Fig. 1:**
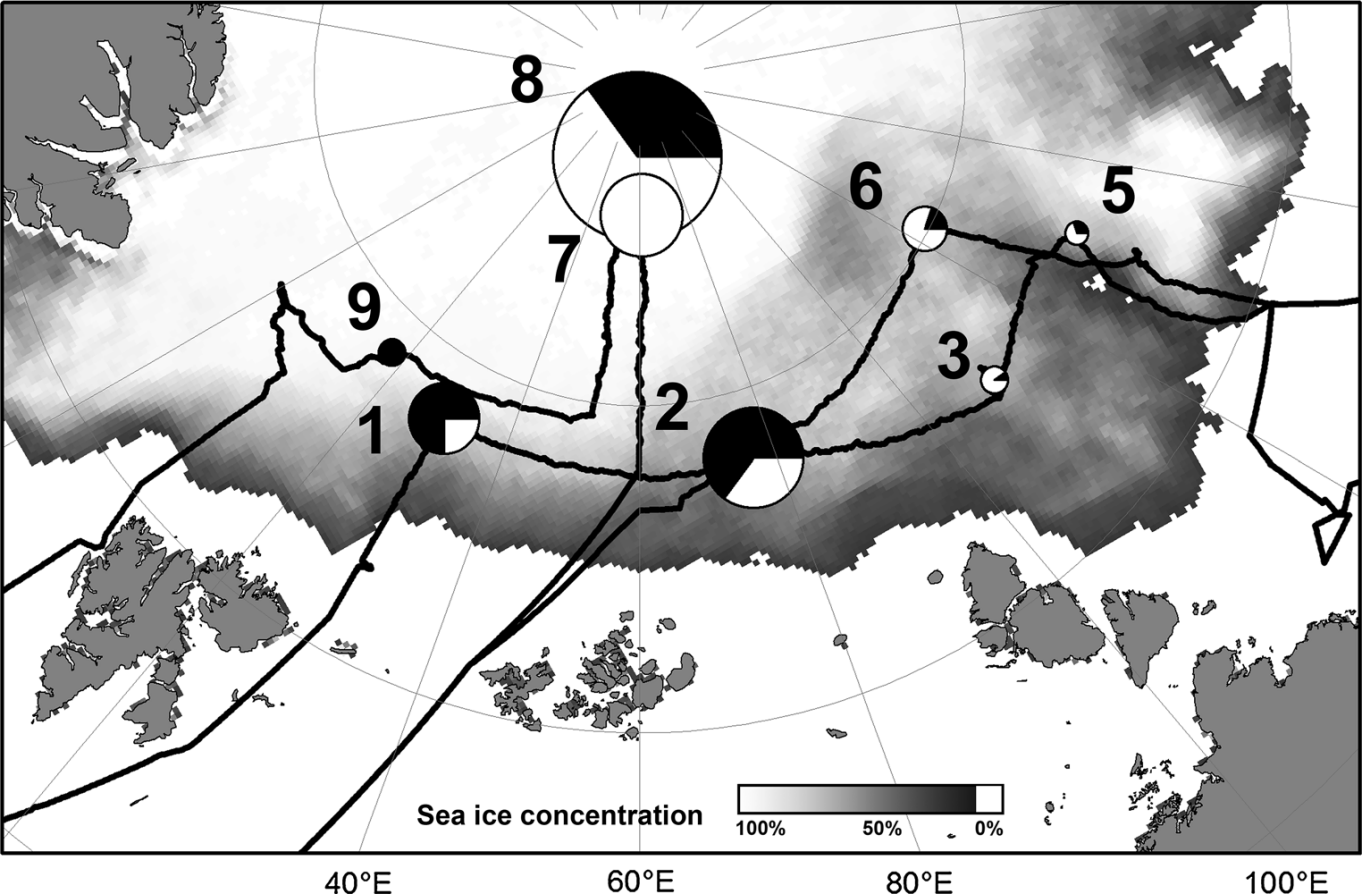
Map of the IceArc expedition cruise track and positions of ice stations where under-ice algal aggregates were observed with the ROV. The *size of the circles* represents relative aggregate abundance (see Table 3 for absolute values), while the fraction of the two aggregate types as determined from mean aggregate eccentricity is depicted by the pie charts. *White color* stands for the fraction of elongated aggregates, while *black* depicts the fraction of rounded aggregates. Mean sea ice concentration during the cruise period is shown in the background

Sea ice algal aggregate observations were carried out during the IceArc cruise (ARK-XXVII/3) of the German research icebreaker RV Polarstern to the Central Arctic in August and September 2012. Eight ice stations were selected along the retreating sea ice edge, as well as within the Central Arctic pack ice close to the geographic North Pole (Fig. 1). The ice floe of the first ice station was revisited at the end of the cruise, enabling repeated sampling of the same ice floe after its transition from summer melting to autumn freeze-up conditions.

We used a V8Sii-ROV (Ocean Modules, Åtvidaberg, Sweden), to investigate the algae distribution underneath the sea ice. It was launched through a hole in the ice and operated from a tent directly on the ice floe several hundred meters away from the ship to avoid disturbance from the ship’s thrusters. At each ice station, the ROV achieved a diving time between 6 and 8 h. The setup and operation procedure was similar to the one used in Nicolaus and Katlein (2013) with minor modifications: A Micron Nav (Tritech, Aberdeen, UK) ultra-short baseline (USBL) positioning system provided precise ROV location in a floe fixed coordinate system, while the rear facing Ospray SD-camera (Tritech, Aberdeen, UK) was repositioned to provide upward-looking imagery.

Additional sensors complemented the observations with measurements of the physical properties of the under-ice habitat. Ice draft was calculated as difference between the ROV depth and its distance to the ice measured by the upward-looking DST micron echosounder (Tritech, Aberdeen, UK) altimeter. Spectral light transmittance (320–950 nm) was calculated from continuous synchronous measurements of downwelling irradiance using two RAMSES-ACC spectroradiometers (TriOS GmbH, Rastede, Germany). While one sensor was mounted on the ROV, a reference sensor was placed on a tripod on the ice close to the ROV launch hole. Data processing, calibration, and measurement uncertainties have been described previously (Nicolaus et al. 2010; Nicolaus and Katlein 2013; Katlein et al. 2014).

At station ICE-7, under-ice aggregates were sampled with the ROV using a custom-built sampling device, to evaluate the image analyses. Samples were analyzed for particulate organic carbon (POC) and species composition as described in Assmy et al. (2013).

### Image classification

To quantify the spatial distribution of under-ice algal aggregates from the acquired ROV imagery, we applied a threshold algorithm implemented in MATLAB. We extracted still images (384 × 288 pixels) every 5 s from the videos of the upward-looking camera using the command line tool *ffmpeg*. Extracting the images every 5 s from the video overcomes problems with multiple detection of the same aggregates in consecutive images in most cases. To account for inconsistent lighting at the image edges, and to mask out the overlay data display, we cropped the image to obtain undisturbed RGB images of 250 × 200 pixels. Analysis of image histograms showed that aggregates could be detected well with a threshold value, by selecting all pixels with a value between 0 and 100 (out of maximal 255) in the green channel as aggregates. Thus, images were converted into binary images for further analyses.

All images taken at a depth >5 m and at ROV tilts >10° were automatically discarded. Images where ice structures or other objects were detected as aggregates and images where clearly identifiable aggregates were not detected by the algorithm were manually discarded. In the case where several close-lying aggregates were mistaken for a larger one, the image was excluded from shape and size analysis. In total, 11,000 images out of 23,800 images were used for further analysis. These images were on average taken at a distance of one meter from the ice underside and resulted in a spatial resolution of 4–5 mm.

Two-dimensional aggregate properties such as perimeter and area as well as minor axis and major axis of a fitted ellipse were calculated for each individual aggregate. For all aggregates covering more than 10 pixels of the image, shape parameters such as eccentricity, circularity, and the equivalent circular diameter were derived. The measurements were transformed from pixel units to real units using the distance to the ice measured by the altimeter and a laboratory calibration of the camera. This image registration to true geometric units also enabled us to determine aggregate abundance per square meter as the number of aggregates in the image divided by the area surveyed by the image. Uncertainties in the distance to the ice, ROV-tilt and lens distortion result in <15 % uncertainty in aggregate size measurements.

The detection algorithm worked well in most situations, but due to its simple nature, detection was problematic in some cases, and 54 % of the images had to be excluded from the analysis. This was mostly related to inhomogeneous backlighting within the image at the transition between different ice features, where dark features in the ice were misinterpreted as aggregates. In addition, the differentiation of aggregates from small-scale ice structures and air bubbles can be ambiguous even to a trained observer verifying the detection. Finally, aggregates that are not dense enough or too small to leave a significant signature in the pixels green value remain undetected.

To account for multiple sampling due to overlapping ROV tracks in some areas (e.g., in the vicinity of the ROV launch hole), all obtained parameters were gridded on a regular grid with 3 × 3 m cell size, corresponding to the maximal uncertainty in the ROV position. All available images within a grid cell were selected, and the average of all measurements from these pictures was assigned to the grid cell.

### Biomass estimation

To estimate aggregate biomass, the two-dimensional distribution deducted from the images was converted into three-dimensional volumetric information, including assumptions about the aggregate shape. While the assumption of a uniform algal layer thickness is more applicable to typical ice algal bottom layers in spring, we chose to represent the typically rounded aggregates by compact spheres. The diameter of the aggregate was determined as the equivalent circular diameter of the connected pixel region from the regions area.

We used different methods to obtain an estimate of the aggregate volume per area. Comparison between the different approaches enables us to evaluate the accuracy of our biomass estimates and the disadvantages of individual approaches. Names given in parentheses identify the different approaches later in the text.

For a first approach, aggregate volume was calculated for every detected aggregate. The single aggregate volumes were then added up and divided by the survey area (“aggregate list”). This approach does not take into account multiple sampling of some aggregates due to overlapping ROV tracks and should thus result in an overestimate of total biovolume.

In a second approach, the aggregate volume *V* was calculated using abundance *a* and diameter *d* values averaged over all images (“global mean”):

1 $$V = a \times \frac{4}{3}\pi \left( \frac{d}{2} \right)^{3}$$

As sphere volume is dependent on the third power of diameter (Eq. 1), this approach was repeated with a median diameter instead of average diameter, to avoid overestimation of biovolume for large, likely not spherical, aggregates (“global median”).

To avoid influences from multiple sampling due to overlapping ROV tracks, the same calculation was repeated with average and median values obtained after gridding of the results (“gridded mean” and “gridded median”). The “gridded median” approach was also used in Assmy et al. (2013).

As larger rounded aggregates can occur in some parts of the survey areas, they can contribute significantly to biomass that gets lost when averaging over many grid cells. Thus, in a last approach, biovolume was calculated from abundance and diameter in each grid cell separately and averaged afterwards (“raster cells”). In the case where aggregates deviate substantially from a spherical shape, this approach can significantly overestimate biovolume.

To convert the estimated biovolume to carbon content, we used the measured carbon content of 390 mg C L^−1^ of two aggregates of known volume from ice station ICE-7 (Assmy et al. 2013).

### Patchiness and size distribution

To analyze the spatial patchiness of the aggregate distribution, we used the index of mean crowding and Lloyds Index of Patchiness (Lloyd 1967; George 1981; Gutt et al. 1991) on the gridded data.

The size distribution of aggregating particles is often described using a power law

2 $$f(d) = cd^{b}$$

with *d* the diameter, *b* the characteristic slope and a normalization constant *c*. The characteristic slope varies for different types of phytoplankton usually around a value of −3 (Alldredge and Gotschalk 1989; Guidi et al. 2009). Equation 2 was used to fit the aggregate size distributions obtained from the list of all detected aggregates and to determine the value of the characteristic slope. The characteristic slope and size distributions were interpreted in comparison with the marine particle aggregation model from Jackson (1990).

## Results

### Sea ice conditions

During the cruise, we observed a variety of sea ice conditions. While the first two stations located in the transpolar drift were comprised of dense first-year ice with a thickness of 1.0–1.5 m, the area of stations ICE-3 to ICE-6 was dominated by extremely rotten sea ice with a thickness of less than 1.0 m in an advanced melting stage. Later during the cruise, we observed freezing conditions, with ice covers of several centimeters forming on the melt ponds. Stations ICE-7 and ICE-8 in the central pack ice consisted of multi-year ice with a level ice thickness of up to 1.8 m. In contrast to the previous stations along the ice edge, the ice was less deteriorated by melt, and we observed the first snowfall in mid-September. The repeated visit to the first floe during ICE-9 was characterized by the fall freeze-up, with a snow cover of about 10 cm and refrozen ponds. In contrast, the topography of the ice underside had not changed dramatically. All observations were made in late summer at the end of the productive season. Thus, we can assume that algal biomass in general was low as compared to spring season, and in particular no ice algal layer was observed at the ice bottom.

### ROV observations

ROV surveys underneath sea ice using upward-looking video imagery are a useful tool to quantify the abundance and spatial distribution of ice algal aggregates. The upward-looking imagery resulted in a continuous observation along the dive tracks, covering a representative fraction of the variability in summer sea ice conditions in the investigated region. The large spatial coverage achieved during rather short station time is of great advantage in mapping the patchy under-ice ecosystem without bias towards sites with more abundant biomass. Detailed analysis of the upward-looking video imagery enabled us to quantify the amount and distribution of ice algal aggregates. Aggregate coverage for our investigated sites varied between 0.003 and 0.163 %, with an average of 0.04 %, while the abundance varied between 0.3 and 16.0 aggregates m^−2^, with an average of 3.4 aggregates m^−2^ (Table 1). A comparison of our new and improved image processing analysis with previous studies using smaller data sets to estimate aggregate coverage and abundance is shown in Table 1. Subsequent analysis of several subsets of the entire dataset revealed that, due to the high spatial patchiness of the aggregates, the result was to some extent dependent on the area covered by the survey. While Assmy et al. (2013) analyzed only data from ice stations ICE-1 and ICE-2, Boetius et al. (2013) analyzed only one representative dive per ice station. As the image registration required manual post-processing, it was not available onboard the ship, and thus Boetius et al. (2013) reported only percent cover instead of aggregate abundance or biomass. When processing capabilities are limited, reasonably good estimates of percent cover can be achieved with the analysis of just one ROV transect if it is selected as representative from all available dives by the ROV pilot. Nevertheless, some transects differ significantly from the rest of the survey area.

**Table 1:** Comparison of aggregate percent coverage and abundance retrieved from different image treatment approaches using varying subsets of the dataset

Ice station	ICE-1	ICE-2	ICE-3	ICE-5	ICE-6	ICE-7	ICE-8	ICE-9
% Coverage								
Boetius et al. (2013) (one dive per station)	0.04	0.19	<0.001	0.04	0.03	0.55	0.13	–
Assmy et al. (2013) (all station dives)	0.01	0.03	–	–	–	–	–	–
This study (full dataset, improved processing)	0.026	0.062	0.005	0.003	0.008	0.163	0.093	0.004
Abundance (agg m^−2^)								
Boetius et al. (2013) (one dive per station)	–	–	–	–	–	–	–	–
Assmy et al. (2013) (all station dives)	0.79	5.06	–	–	–	–	–	–
This study (full dataset, improved processing)	2.85	5.69	0.48	0.32	1.13	3.85	16.07	0.41

**Fig. 2:**
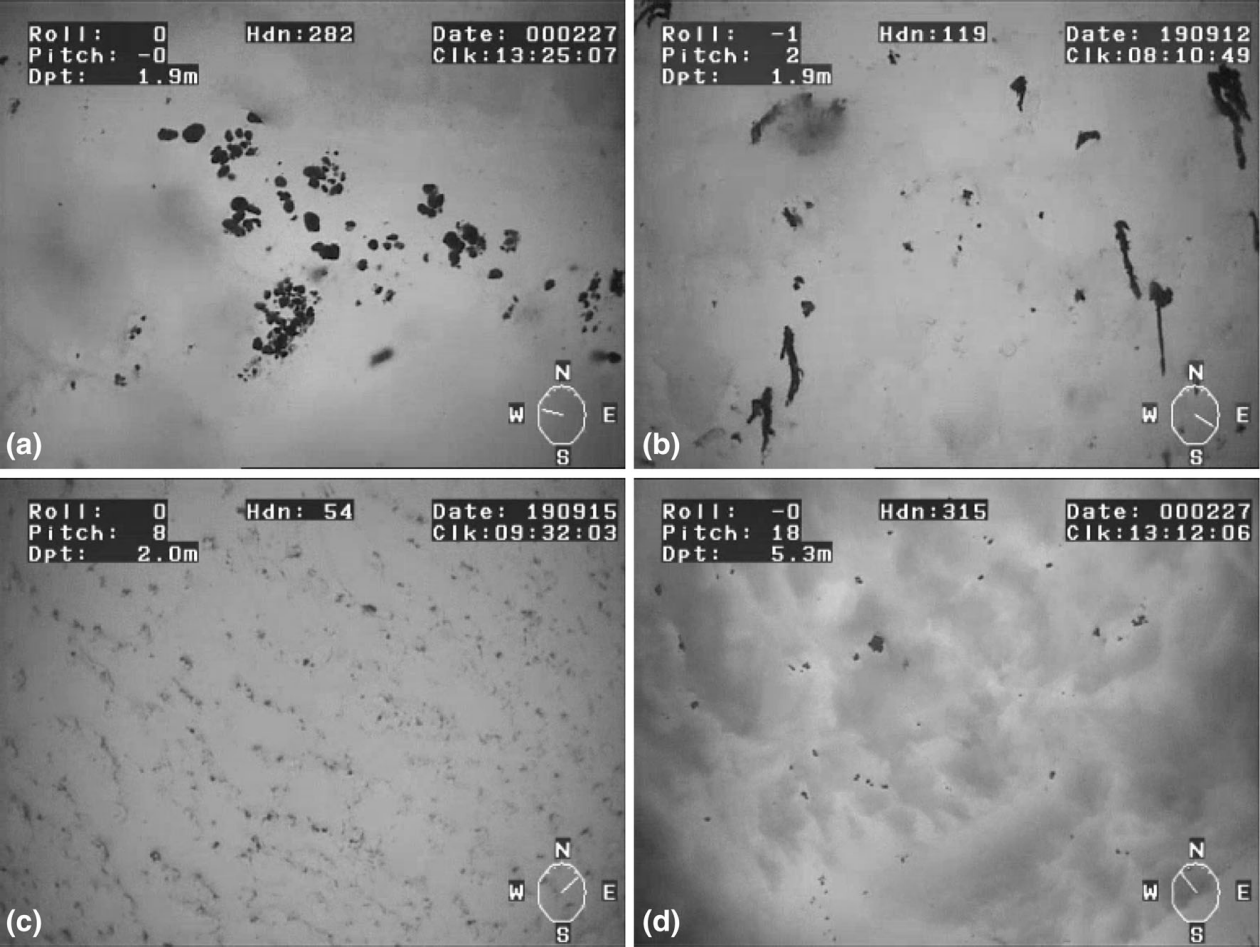
Example images from the upward-looking ROV camera: **a** rounded aggregates formed by pennate diatoms, **b** filamentous aggregates of *M. arctica*, **c** a regular cover of small aggregates close to the detection limit of the method, **d** a tilted view from greater depth shows that aggregates are often trapped within dome-like or rough ice structures

Aggregates were observed both under first-year and under multi-year sea ice. Under-ice aggregates were found to be either free-floating, rounded masses dominantly formed by pennate diatoms (Fig. 2a), or elongated filamentous strings attached to or floating underneath the sea ice and composed of *M. arctica* (Fig. 2b). Details of the composition, development and fate of these aggregates have been described elsewhere (Assmy et al. 2013; Boetius et al. 2013; Fernández-Méndez et al. 2014).

### Spatial distribution on floe scale

**Fig. 3:**
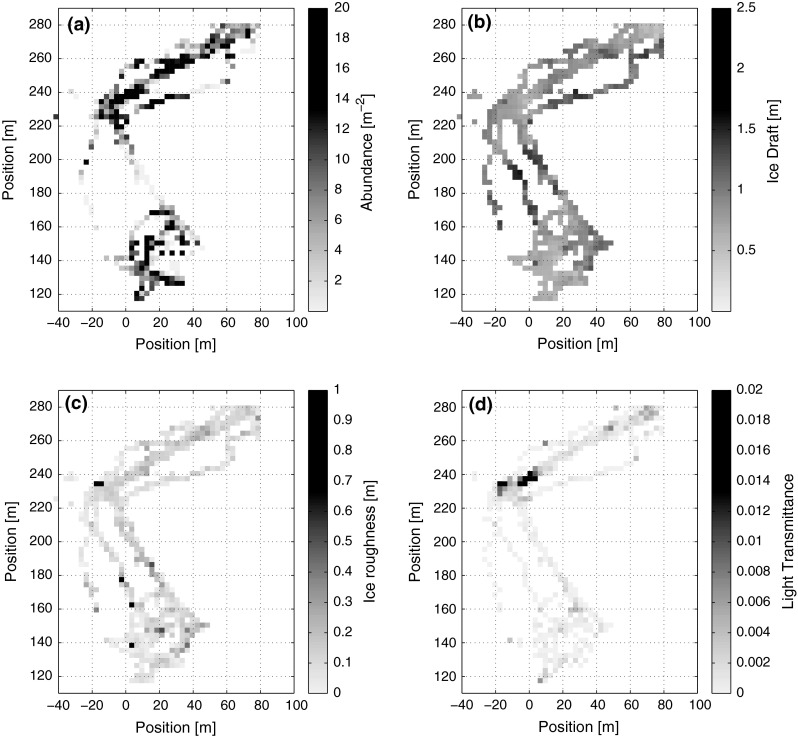
Spatial distribution of aggregates (**a**), ice draft (**b**), ice roughness (**c**), and light transmittance (**d**) on station ICE-8. Distribution maps of the other stations can be found in the electronic supplement (Online Resource 1). Positions are given in a floe fixed coordinate system relative to the ship’s GPS receiver

Maps of aggregate distributions were constructed from the results of the image analysis. A representative example can be found in Fig. 3 and all other stations in Online Resource 1. The aggregate distribution exhibited high variation, which is indicated by high values of Lloyd’s index of patchiness, especially for stations with low aggregate abundance. The aggregate distribution is very patchy, and abundances vary from vast empty stretches to accumulations with peak detections of up to 200 aggregates m^−2^ on short distances of only tens of meters.

According to the visual impression from upward- and forward-looking ROV cameras, most of the aggregates were floating freely up against the underside of the sea ice. The buoyant status of the aggregates could be assessed after detachment from their original position by thruster disturbance. After disturbance, they again slowly rose up against the ice-water interface, indicating slightly positive buoyancy. Due to this positive buoyancy, most of them were situated in dome-like structures with a depth of just a few centimeters (Fig. 2d).

**Fig. 4:**
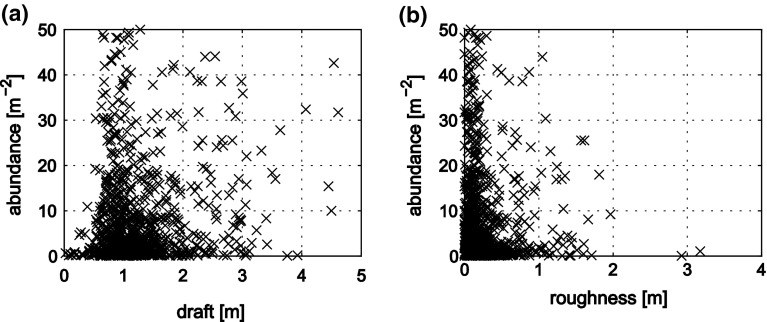
Dependence of aggregate abundance on **a** sea ice draft and **b** sea ice roughness

While one might expect a relation of the aggregate distribution to light availability under sea ice, due to better conditions for growth and floatation (Fernández-Méndez et al. 2014), no direct correlation of the spatial distribution of light transmittance and the aggregate distribution was found. As indicated by the visual observations, the only relation of aggregate abundance was found when comparing it to maps of ice thickness and roughness (Fig. 3 and Online Resource 1). High aggregate abundances often occurred at the boundaries of ridge keels and especially in level ice with moderate roughness (Fig. 4). Pressure ridges themselves did not host significant aggregate accumulations.

### Biomass estimation

Results of the biomass estimates obtained by the different calculation approaches are shown in Table 2. While the different methods yielded a consistent picture of relative aggregate biomass at the different stations, they exhibited large quantitative differences. Biomass estimates spanned up to three orders of magnitude from <0.01 to 20.45 mg C m^−2^ even though they were derived with only slightly varying algorithms from the same dataset.

**Table 2:** Biomass estimates obtained by different approaches

Ice station	ICE-1	ICE-2	ICE-3	ICE-5	ICE-6	ICE-7	ICE-8	ICE-9	Mean
Aggregate volume (ml m^−2^)									
Global mean	0.4	2.1	0.02	0.03	0.04	10.4	1.1	0.03	1.8
Global median	0.2	0.5	<0.01	0.01	0.01	4.8	0.5	0.01	0.7
Gridded mean	2.5	6.0	0.7	0.3	0.4	16.8	6.5	0.3	4.2
Gridded median	0.9	1.4	0.5	0.1	0.2	21.9	4.1	0.3	3.7
Raster cells	5.2	16.3	4.7	1.6	3.0	38.6	6.9	1.7	9.7
Aggregate list	2.2	20.4	0.2	0.5	0.7	52.5	6.9	0.4	10.5
Carbon content (mg C m^−2^)									
Global mean	0.2	0.8	0.01	0.01	0.02	4.0	0.4	0.01	0.7
Global median	0.1	0.2	<0.01	<0.01	<0.01	1.9	0.2	<0.01	0.3
Gridded mean	1.0	2.3	0.3	0.1	0.1	6.5	2.5	0.1	1.6
Gridded median	0.4	0.5	0.2	0.03	0.07	8.5	1.6	0.1	1.4
Raster cells	2.0	6.4	1.8	0.6	1.2	15.0	2.7	0.7	3.8
Aggregate list	0.8	8.0	0.1	0.2	0.3	20.4	2.7	0.1	4.1

“Global” refers to averages over all images with valid information, while “gridded” refers to averages determined after spatial gridding of the results. “Mean” and “median” refer to whether mean or median diameters were used in the calculation. “Raster cells” refers to biomass calculation within the spatial grid cells before averaging over the survey area, while “aggregate list” refers to a calculation based on the list of all aggregate detections. The approaches “gridded median” and “raster cells” should provide the most reasonable estimates

### Aggregate properties

Mean properties of the detected aggregates are given together with the environmental parameters in Table 3. Mean aggregate diameters ranged from 2.1 to 4.1 cm, and mean abundances ranged from 0.3 up to 16.0 aggregates per m^2^. Mean aggregate eccentricities ranged from 0.76 to 0.88. The minima and maxima of observed eccentricities ($\varepsilon_{ \hbox{min} }$ and $\varepsilon_{ \hbox{max} }$) coincided with the visual observation of sole occurrence of rounded and filamentous aggregates. Thus, we deduced aggregate-type fractions ($f_{\text{spherical}}$ and $f_{\text{elong}}$) from a linear mapping to the eccentricity value $\varepsilon$ with $f_{\text{spherical}} = \frac{{\varepsilon - \varepsilon_{ \hbox{min} } }}{{\varepsilon_{ \hbox{max} } - \varepsilon_{ \hbox{min} } }}$ and $f_{\text{elong}} = 1 - f_{\text{spherical}}$ (Fig. 1). While elongated filaments, corresponding to *M. arctica* aggregates, dominated the stations close to the Laptev Sea (ICE-3, ICE-5; ICE-6) and in the central pack ice (ICE-7, ICE-8), the stations further down the transpolar drift towards Fram Strait (ICE-1, ICE-2, ICE-9) that were dominated by rounded aggregates formed by pennate diatoms.

**Table 3:** Environmental parameters at the ice stations and average aggregate properties

Ice station	Units	ICE-1	ICE-2	ICE-3	ICE-5	ICE-6	ICE-7	ICE-8	ICE-9
Polarstern station #		PS80/224	PS80/237	PS80/255	PS80/323	PS80/335	PS80/349	PS80/360	PS80/384
Latitude	°	84.00	83.95	82.86	82.88	85.06	87.93	88.83	84.35
Longitude	°	30.00	76.85	109.86	130.76	122.52	60.95	58.53	17.73
Date in 2012		10 Aug	15 Aug	20 Aug	5 Sep	8 Sep	19 Sep	22 Sep	29 Sep
Water depth	m	4,300	4,300	4,290	4,020	4,000	3,250	4,090	3,700
Sea ice concentration	%	80	80	70	60	50	100	100	100
Sea ice thickness	m	1.0	1.3	0.9	0.8	0.7	1.6	1.8	1.2
Sea ice type		FYI	FYI	FYI	FYI	FYI	MYI	MYI	FYI
Abundance	Agg. m^−2^	2.8	5.6	0.4	0.3	1.1	3.8	16.0	0.4
Diameter (median)	cm	2.1	2.0	3.4	2.1	1.6	3.5	2.0	1.5
Diameter (mean)	cm	2.5	2.9	4.1	2.9	2.2	4.0	2.4	2.1
Circularity	–	0.79	0.77	0.66	0.70	0.68	0.64	0.71	0.84
Eccentricity	–	0.79	0.81	0.89	0.87	0.85	0.88	0.87	0.76
Index of Patchiness	–	11.5	9.2	67.4	17.9	74.0	3.1	3.3	5.2
Distance to ice edge	km	180	190	380	300	510	600	700	320
Distance to Laptev sea	km	1,450	910	580	490	740	1,210	1,210	1,560
Melt water^a^	m	0.5	0.7	0.7	2.3	2.2	0.8	0.9	–
Deep-sea algae cover^a^	%	0	0.003	1.3	0.5	0.8	2.2	10.4	–
slope of size distribution	–	−1.9	−2.2	−1.3	−1.7	−2.3	−2.2	−3.1	−1.6
Slope (*Ø* > 2 cm)	–	−3.0	−3.0	−0.6^b^	−1.2^b^	−2.0^b^	−3.2	−3.9	−1.0^b^

^a^Data from Boetius et al. (2013)

^b^ *R* ^2^ < 0.8

**Fig. 5:**
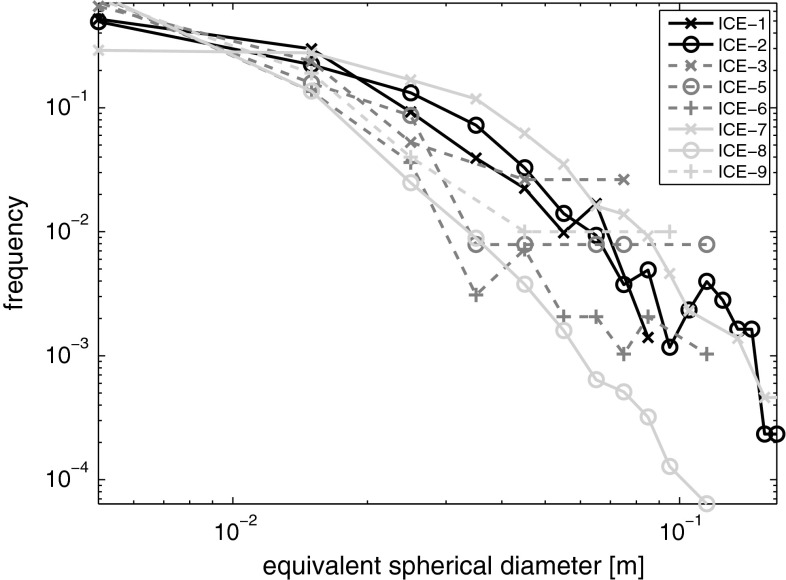
Aggregate size distribution on the different ice stations. Size distributions that flatten out towards big aggregates are shown by *dashed lines*

The size distribution of algal aggregates obtained from the image analysis for all stations generally followed the expected power law (Fig. 5). The characteristic slope obtained from power law fitting ranged from −1.3 to −3. It showed a correlation to the latitude of the ice station (*p* = 0.014). However, some important deviations between the different ice stations could be recognized. A distinct and unexpected feature was that the size distribution on ice stations 3, 5, 6, and 9 flattened out towards larger aggregates indicating enhanced buoyancy.

## Discussion

### Limitations of aggregate detection

Even though our analysis of upward-looking ROV images is currently the best available method for aggregate quantification on larger scales, one needs to keep in mind some limitations of the method. Firstly, the method is only able to detect macroscopic aggregates bigger than a few millimeters floating directly underneath the sea ice. This relatively high detection limit leads to an underestimation of the total algal aggregate biomass, but might be irrelevant in light of the huge range of biomass estimates caused by the different estimation algorithms. Secondly, close-lying aggregates that are detected as a single one, as well as aggregates with a strong deviation from the spherical shape, can lead to an overestimation of total aggregate volume. To reduce this effect, we excluded clumped aggregates from the analysis. This affects <5 % of the detected aggregates. Thirdly, aggregates are often located in transition zones between different ice types, which at the same time are often discarded during image processing, as the darker background of the thicker ice type gets classified as aggregates by the threshold algorithm. Overall, our method as used in this study might be rather underestimating ice algal aggregates. Future studies could thus benefit from a more sophisticated image classification technique and machine learning for automation of the detection.

### Patchiness and biomass estimates

The patchy spatial distribution of algal aggregates makes accurate, large-scale estimates of the aggregate biomass very challenging. As our results show, not only the choice and range of sampling sites, but also the method of estimating biomass from the data, may heavily impact the estimates. Consequently, small-scale surveys of the under-ice ecosystem such as diver observations are influenced by the choice of sampling sites. When comparing different surveys from ROVs and diver studies, differences of several orders of magnitude might simply arise due to differences in data processing and the size of the survey area. These differences can be even more dramatic when compared to results obtained by classical ice coring that do not usually capture algal aggregates. Larger-scale surveys tend to give a more realistic estimate (Assmy et al. 2013), accounting also for large areas empty of aggregates, which could be under-represented in spot measurements. Hence, values found in the literature should be used with caution for upscaling calculations. As estimates of aggregate biomass are highly dependent on the diameter of the aggregate, approaches that resolve spatial differences in aggregate properties (“raster cells”) and account for multiple sampling (“gridded median”) should give the most reliable results (Table 2). When considering only these two algorithms, which very likely are the most reliable ones, the average aggregate biomass at our investigated sites and season accounts for 0.1–6 mg C m^−2^.

Despite improvements in their quantification, algal aggregates will be difficult to include in ecosystem models, as low areal average biomass cannot describe their patchy distribution and thus their role as hotspots of biological activity (Assmy et al. 2013; Fernández-Méndez et al. 2014).

In comparison with our results, the study of Ambrose et al. (2005) shows a much higher percent coverage of 40–90 % of algae. This is due to the fact that the study was conducted much earlier in the season (June) on the shelf, and apart from aggregations also included the thin algal layer at the ice bottom in the analysis. Percent cover as presented in Ambrose et al. (2005) is a challenging proxy of total biomass due to the variable three-dimensional appearance of ice algae and aggregates. While in our dataset percent cover was only weakly correlated to aggregate abundance (*p* = 0.16), it was a better indicator of the total aggregate volume (*p* = 0.001–0.003 for the different algorithms). Aggregate abundance measured using ROV observations off Greenland in June/July varied between <1 and 50 aggregates m^−2^ (Gutt 1995) compares well to our study with an average abundance of <1 up to 16 aggregates m^−2^ with peak detections in a few images of maximal 200 aggregates m^−2^. Recent diving investigations from an ice floe in the Fram Strait also revealed abundances of 6.3 ± 3.1 aggregates m^−2^ (Glud et al. 2014). While abundance values compare well, Glud et al. (2014) derived biomass estimates of up to 2.94 mg Chl *a* m^−2^. This significantly exceeds our estimates of <0.01 to 0.19 mg Chl *a* m^−2^. The large difference can be explained by the seasonal cycle of ice algal development, including seasonal variability in carbon to chlorophyll ratios, as well as differences in methodology.

### Spatial distribution

**Fig. 6:**
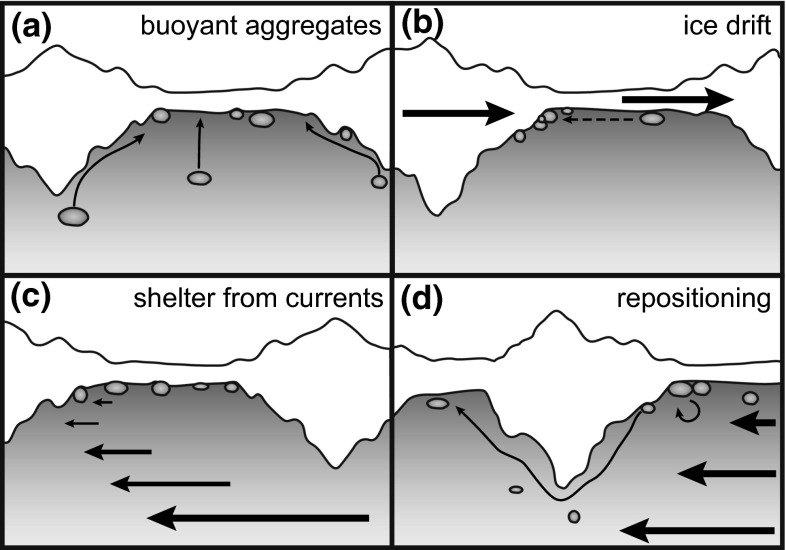
Summary of the four main physical processes governing the spatial distribution of aggregates: **a** Buoyant aggregates are floating up against the ice and accumulate in level ice and dome-shaped structures; **b** during ice drift, pressure ridges skim through the water and can press the aggregates towards ridge edges; **c** location of the aggregates in the level ice and dome-shaped structures provides shelter from under-ice currents; **d** When these get stronger, aggregates get transported further by turbulent water motion

Our results show that the spatial distribution of under-ice algal-aggregate biomass is mostly dependent on the topography of the ice underside and the hydrodynamic regime. Ridge edges, dome-like structures, pockholes and small-scale roughness trap the loosely floating aggregates, leading to accumulations of aggregates in such topographic features. In contrast, pressure ridges themselves, did not host aggregate accumulations due to their large drafts, but rather acted as barriers hindering further aggregate movement. The aggregate distribution is likely very dynamic and can easily be affected by changing ice relative currents, such as strong winds or tides (Assmy et al. 2013; Glud et al. 2014). During such events, algal aggregates can get suspended in the mixed layer and drift along the ice until they get trapped again in the next ice feature. These main mechanisms of physical aggregate redistribution are summarized in Fig. 6. This solely physical mechanism differs strongly from the spatial distribution patterns of actively swimming zooplankton, which can use pressure ridges as a shelter (Gradinger et al. 2010). Also typical habitat properties determining organism distribution such as light availability did not explain the aggregate distribution, as the individual aggregate cannot position itself actively. Its position is determined passively by a complex hydrodynamic interaction between buoyancy, under-ice currents and turbulence, as well as the topography of the ice underside. Nevertheless, a wide range of habitat properties like the availability of light and nutrients as well as grazing of course influence the growth of sea ice algae, the formation of aggregates, and their fate related to sinking or suspension (Fernández-Méndez et al. 2014). While these factors impact the overall aggregate biomass, and might be responsible for the large biomass variation observed in this and other studies, the spatial distribution of aggregates on floe scale is determined by the topography of the ice underside.

This observed distribution pattern is typical of free-floating buoyant aggregates. While the rounded aggregates, formed by ice algae that aggregate after being flushed out of the brine channels due to summer-melt, are always free-floating, elongated aggregates formed by *M. arctica* have been observed both free-floating and attached to the ice. As we observed mostly free-floating aggregates, the spatial distribution of algal filaments attached to the ice could be different than described here. Nevertheless, we suppose that their spatial distribution is similar, as they also profit from current protection by pressure ridges. Even for attached aggregates, we would not expect a direct relation to the under-ice light field, as higher light penetration, e.g., through melt ponds can often be related to higher meltwater fluxes, and thus more difficult conditions for under-ice attachment.

Aggregate biomass as quantified by abundance or percent cover showed some positive correlation to geographical latitude (*p* = 0.02–0.03), indicating that aggregate biomass is greater within the Central Arctic basin than at the ice edge at the end of the summer. According to the shape analysis (Fig. 1), the fraction of filamentous aggregates of *Melosira arctica* seems to be decreasing with increasing distance from the Laptev Sea. This is consistent with previous observations. Ambrose et al. (2005) and Melnikov (1997) described *M. arctica* as occurring on the shelves of the Arctic, where its spores can get incorporated during ice formation in polynias and be transported into the central basin (Smetacek 1985). The fraction of rounded aggregates composed of pennate diatoms increases towards the ice edge. Syvertsen (1991) described a similar succession of pelagic and ice algal flocs in the Barents Sea, followed by filaments of *M. arctica* towards the central pack ice.

### Implications for carbon export

It was possible to resample the ice floe of ice station ICE-1 (10 August) almost 2 months later on 29 September at the end of the productive season, when light availability was strongly reduced. Assuming that the ice floe was in both cases representative for the area and that thus displacement of aggregates by advection can be neglected, we can deduce some information about the changes in the aggregate distribution during that time period. Along with the decrease in aggregate abundance from 2.8 to 0.4 aggregates m^−2^ and the decrease in median diameter from 2.1 to 1.5 cm, we estimate that 67–94 % of the biomass present during the first sampling disappeared by the end of September. Observations of fresh aggregates on the sea floor indicate that they sank to the deep sea (Boetius et al. 2013), but consumption by zooplankton could have played a role as well (Assmy et al. 2013). The aggregate size distributions of both samplings reveal significant differences in the buoyancy status of the aggregate population. While the size distribution in August resembles a more typical distribution of aggregates prone to sinking (McCave 1984), the size distribution at the end of September levels out towards larger aggregates. This flattening of the size distribution towards larger sizes could be reproduced by deactivating the sinking term in the aggregate formation model from Jackson (1990). It thus indicates that aggregates that are still present in September are buoyant and have so far avoided sinking, while the non-buoyant portion of the aggregate population sank down between the first and second samplings. Analyzing the size distributions, we found a signature of buoyant aggregates mainly in the stations closest to the Laptev Sea shelf edge. In this area, we observed extremely rotten and melting sea ice with favorable conditions for aggregate floatation due to sufficient light available for oxygen production (Glud et al. 2014; Fernández-Méndez et al. 2014).

The theory of particle aggregation also yields critical POC concentrations above which phytoplankton exhibits a high aggregation potential (Jackson 1990). Water column concentrations of 70–100 µg L^−1^ POC from our field sites (Fernández-Méndez et al. 2014) thus imply that with typical under-ice shear rates between 0.001 and 1 s^−1^ (McPhee and Morison 2001), the sticking efficiency must be very high. Accordingly the Kolmogorov length scale of turbulence, describing the length scale at which aggregates are prone to breakup processes, is only 0.2–3 cm. The aggregates must thus be bonded together strongly, avoiding aggregate breakup. This matches previous stickiness estimates (Riebesell 1991; Hansen and Kiorboe 1997) and our observations that the aggregates even withstand thruster wash from the ROV.

The ice algal aggregates described in this study are of extraordinary size, when compared to size-ranges observed in flocculation studies in other seas (Riebesell et al. 1991; Alldredge and Gotschalk 1989). When applying relationships between diameter and sinking speed from the literature (Jackson 1990), aggregates with a diameter of 3 cm will reach the deep-sea floor surprisingly fast within a single day once they lose buoyancy at the surface, potentially due to insufficient light conditions or high respiration rates within the aggregates (Fernández-Méndez et al. 2014). This is consistent with observations of fresh ice algal aggregates in water depths around 4,000 m from Boetius et al. (2013).

We conclude that the spatial distribution of under-ice algal aggregates is mainly governed by the topography of the ice underside. Aggregates float up against any dome-shaped structures, and ridge edges inhibit further movement. Thus, sea ice ridges play an important role in structuring the spatial distribution of ice algal aggregates. On the large scale, filamentous aggregates of *M. arctica* are the dominant aggregate type in the inner part of the Central Arctic pack ice, while closer to the ice edge under melting sea ice, rounded aggregates mainly formed by pennate diatoms dominate. The size distribution of aggregates indicates that at least a portion of them do stay afloat and can get incorporated into the ice during freeze-up. Even though our ROV-based method has proved suitable for providing the first large-scale quantitative estimate of aggregate biomass, it remains difficult to compare biomass estimates to other studies, due to the high patchiness, and uncertainties in both samplings and in particular in different ways of deriving areal average estimates from the observations.

## Electronic supplementary material

Below is the link to the electronic supplementary material.

Supplementary material 1 (PDF 1.60 mb)
