# Supplementary material for: Distribution of algal aggregates under summer sea ice in the Central Arctic
Source: Polar Biol. 2014 Dec 17;38(5):719–31. doi: 10.1007/s00300-014-1634-3 (PMC4525807; doi:10.1007/s00300-014-1634-3)

Electronic supplemental material
Distribution of algal aggregates under summer sea ice in the Central Arctic

Polar Biology

Christian Katlein[1], Mar Fernández-Méndez[1,2], Frank Wenzhöfer[1,2], Marcel Nicolaus[1]

[1]Alfred-Wegener-Institut Helmholtz-Zentrum für Polar- und Meeresforschung

Bussestr. 24, 27570 Bremerhaven, Germany, Christian.Katlein@awi.de

[2]Max Planck Institute for Marine Microbiology, 28359 Bremen, Germany

Fig S1 Map of the spatial distribution of aggregates (a), ice draft (b), ice roughness (c) and light transmittance (d) on station ICE-1.

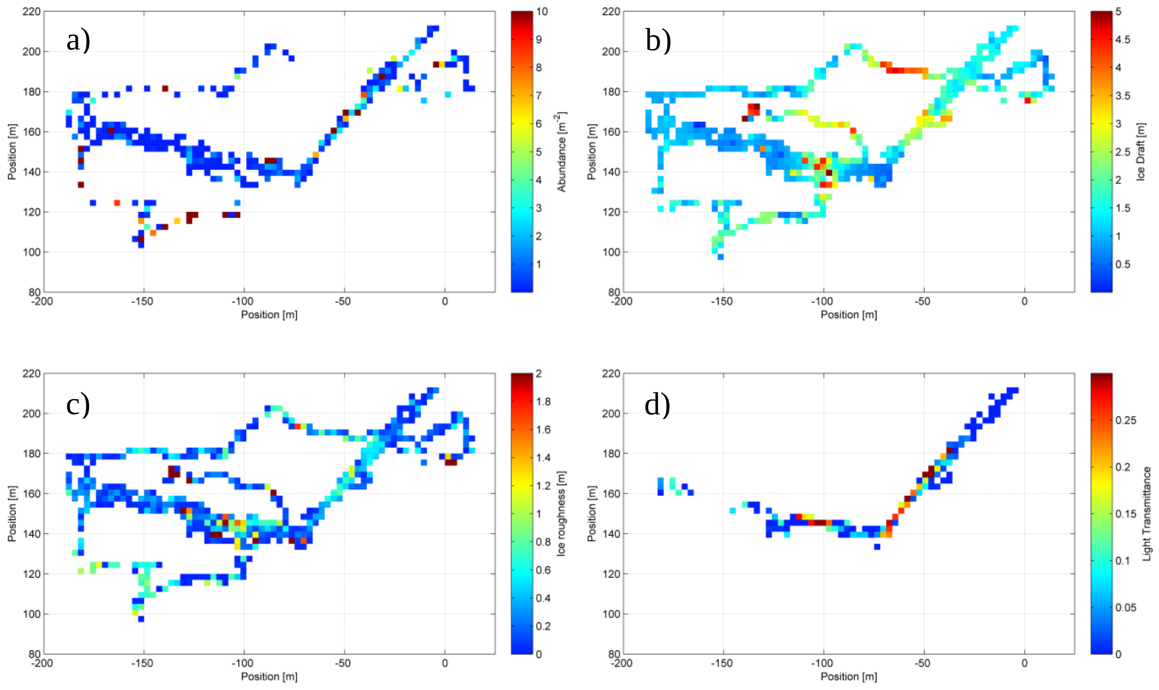

Fig S2 Map of the spatial distribution of aggregates (a), ice draft (b), ice roughness (c) and light transmittance (d) on station ICE-2.

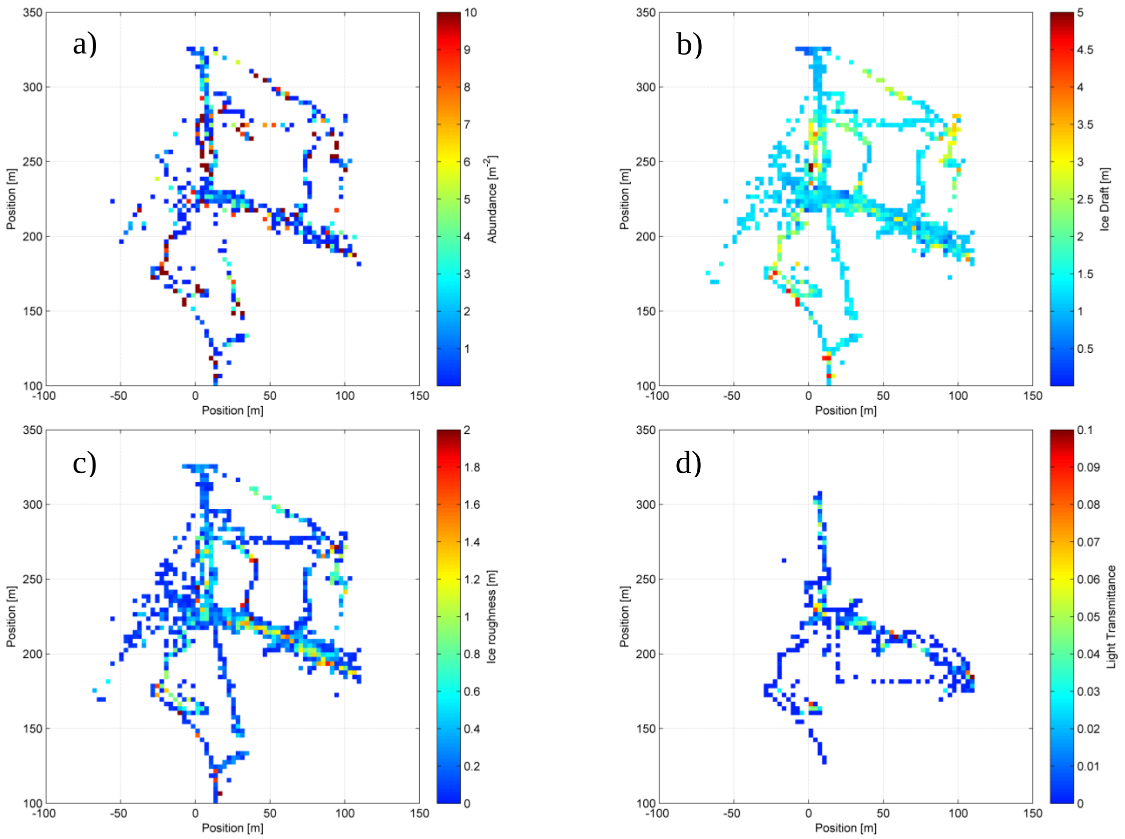

Fig S3 Map of the spatial distribution of aggregates (a), ice draft (b), ice roughness (c) and light transmittance (d) on station ICE-3.

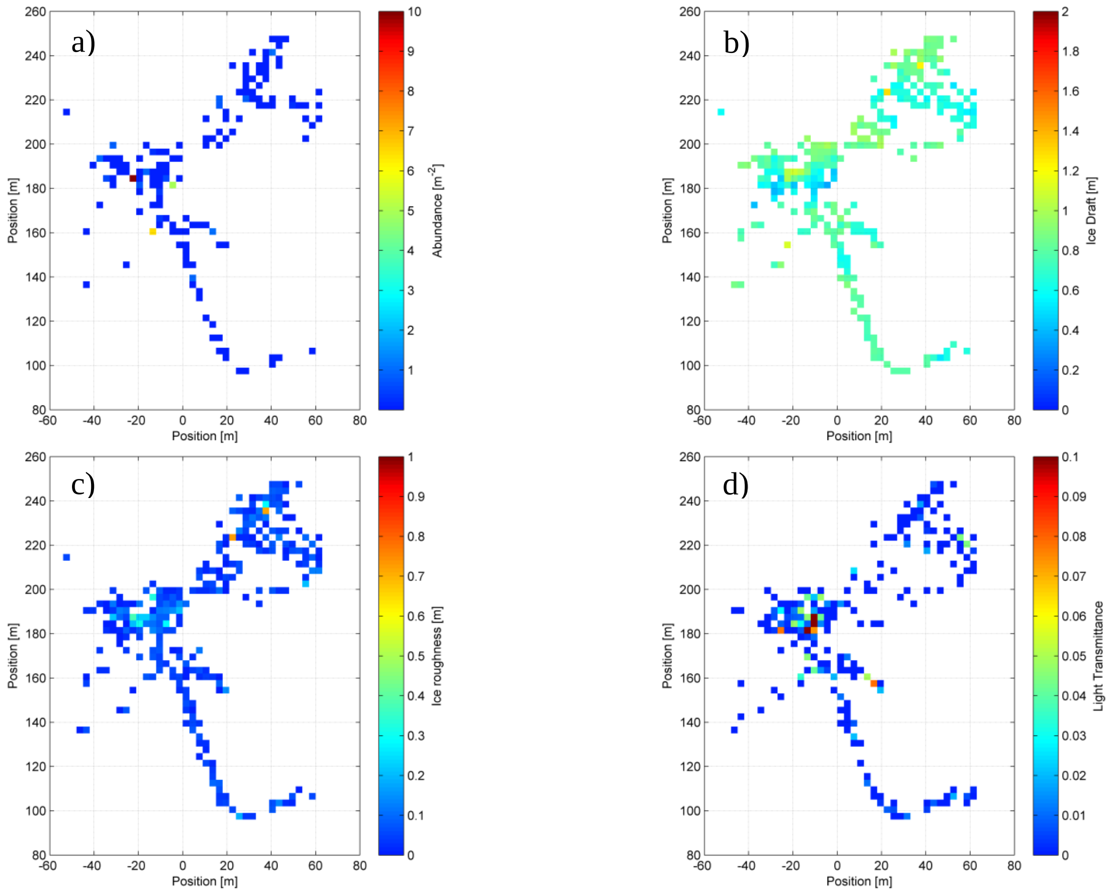

Fig S4 Map of the spatial distribution of aggregates (a), ice draft (b), ice roughness (c) and light transmittance (d) on station ICE-5.

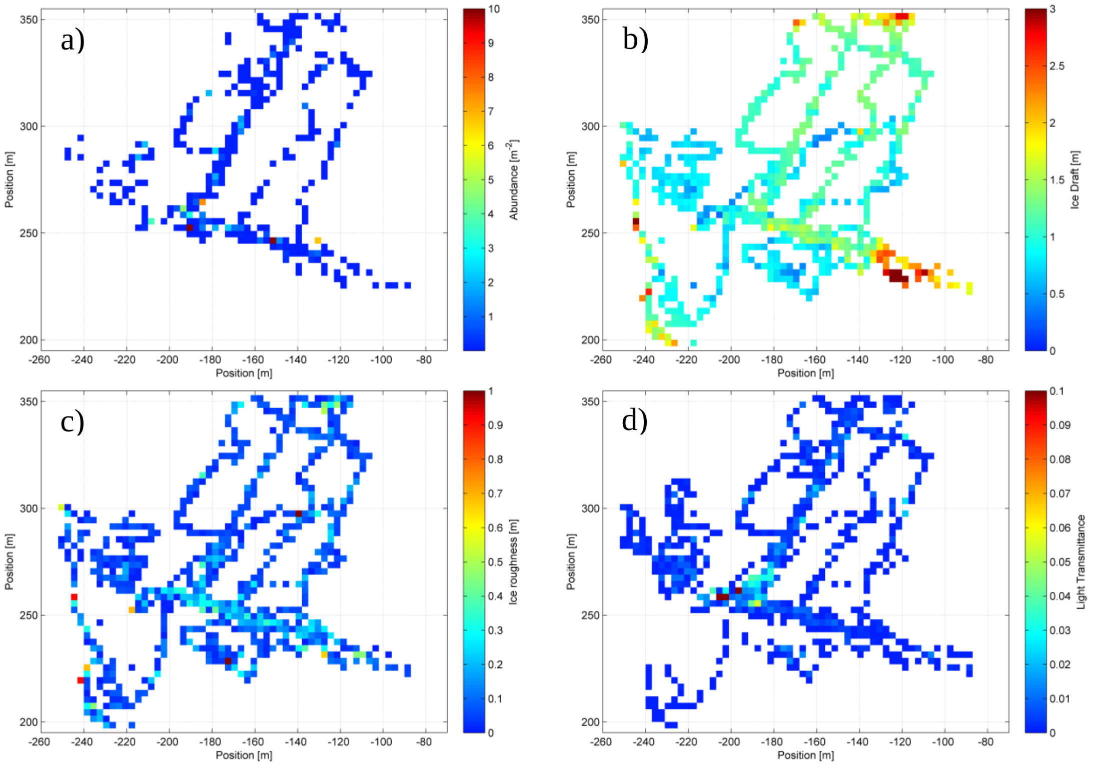

Fig S5 Map of the spatial distribution of aggregates (a), ice draft (b), ice roughness (c) and light transmittance (d) on station ICE-6.

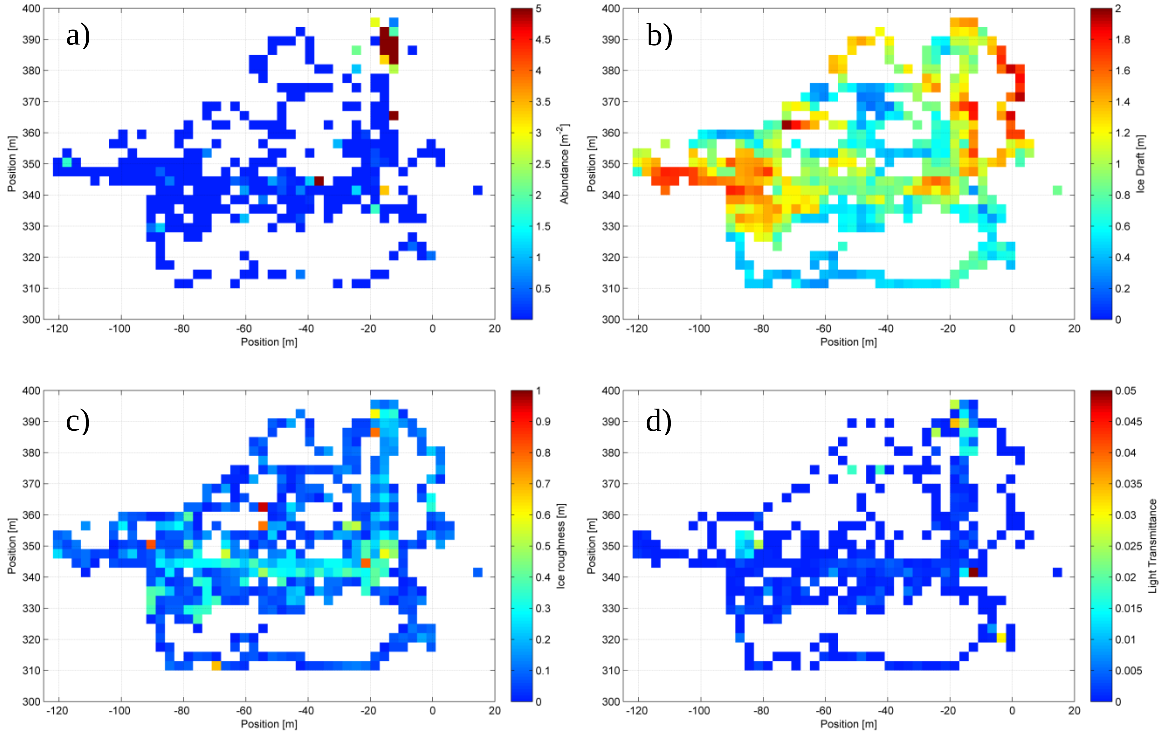

Fig S6 Map of the spatial distribution of aggregates (a), ice draft (b), ice roughness (c) and light transmittance (d) on station ICE-7.

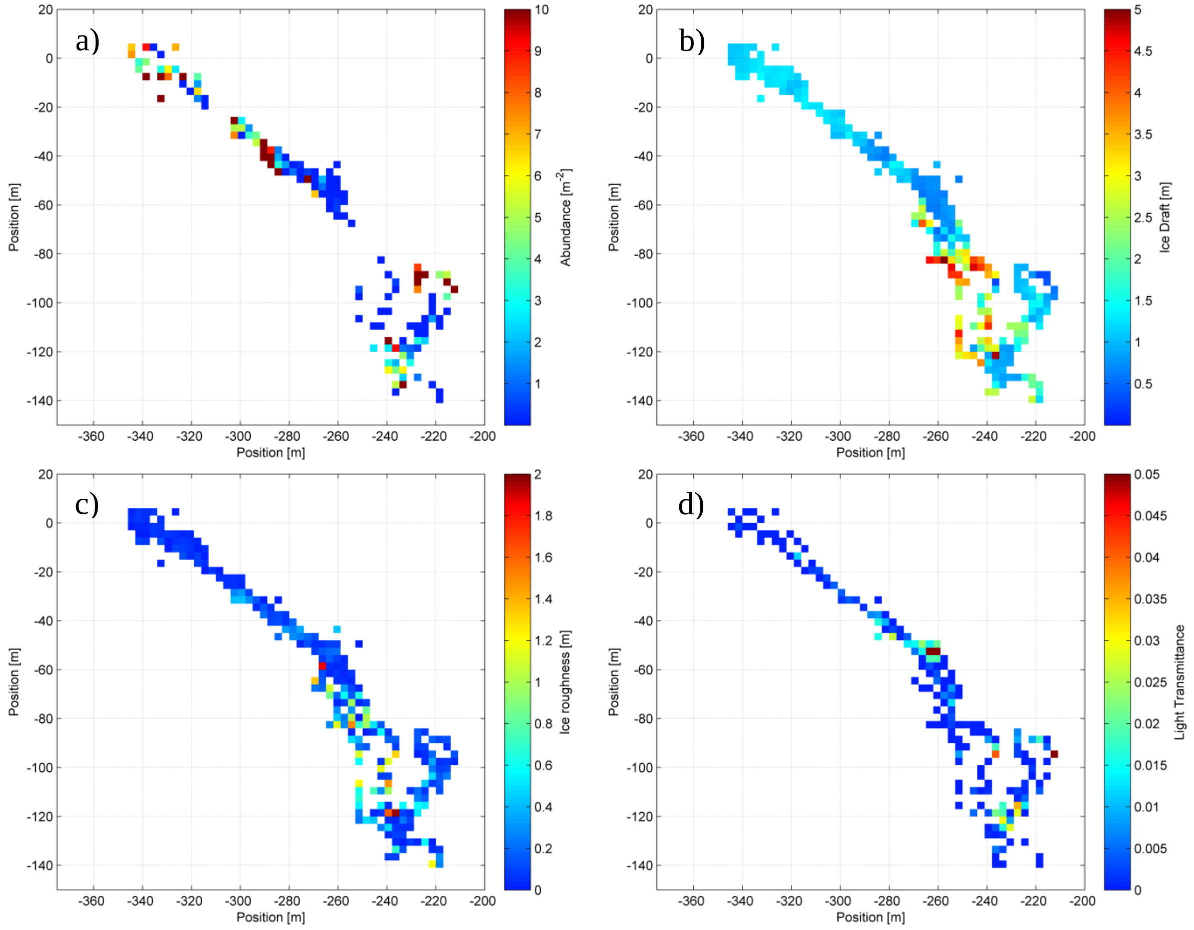

Fig S7 Map of the spatial distribution of aggregates (a), ice draft (b), ice roughness (c) and light transmittance (d) on station ICE-9.

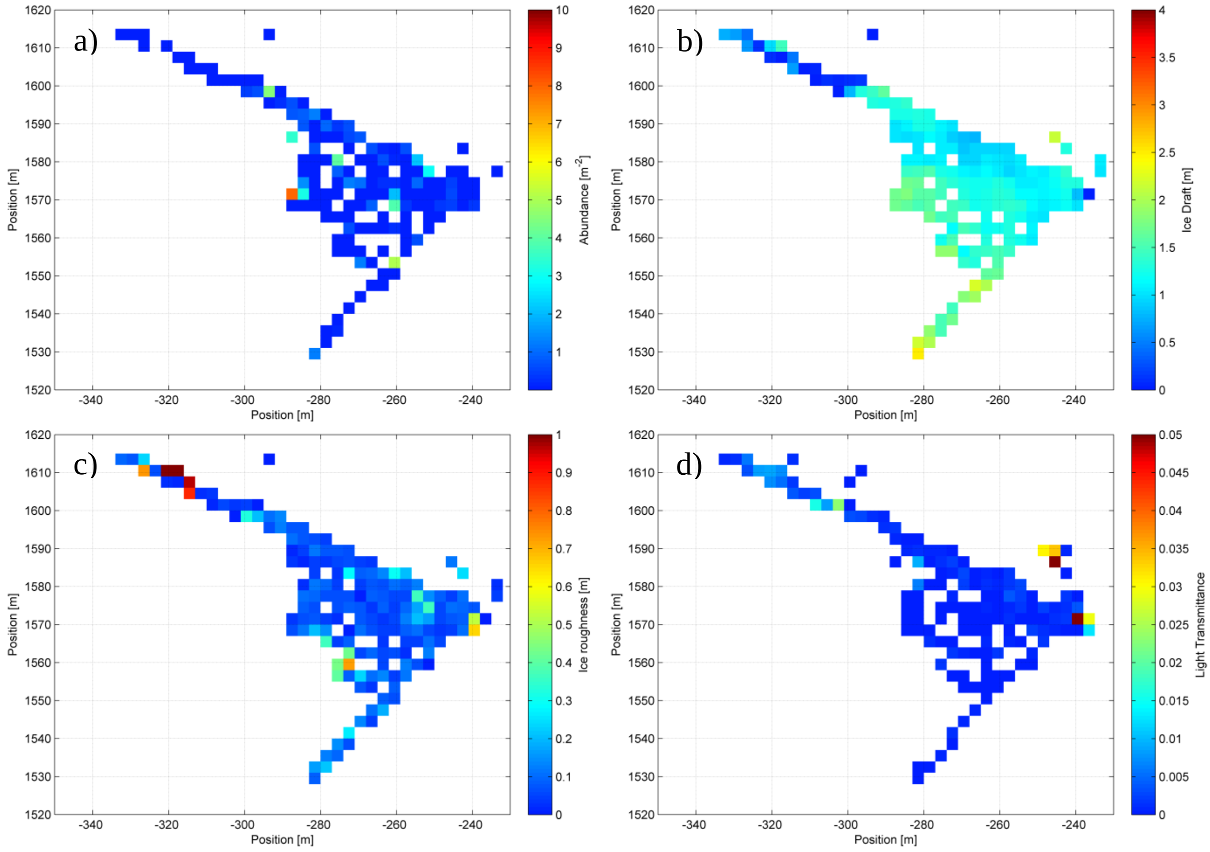

Supplement: Supplementary file 1 — Supplementary material 1 (PDF 1.60 mb) [file 300_2014_1634_MOESM1_ESM.pdf]
